# Pattern recognition receptors and inflammasome: Now and beyond

**DOI:** 10.1016/j.mocell.2025.100239

**Published:** 2025-06-15

**Authors:** SuHyeon Oh, Young Ki Choi, SangJoon Lee

**Affiliations:** 1 Department of Biological Science, Ulsan National Institute of Science and Technology (UNIST), Ulsan, Republic of Korea; 2 Center of Study of Emerging and Re-emerging Viruses, Korea Virus Research Institute, Institute for Basic Science (IBS), Daejeon, Republic of Korea; 3 Graduate School of Health Science and Technology, Ulsan National institute of Science and Technology (UNIST), Ulsan, Republic of Korea

**Keywords:** Immune response, Inflammasome, Inflammatory cell death, Innate immunity, Pattern recognition receptor

## Abstract

Pattern recognition receptors (PRRs) are fundamental to the innate immune system, functioning to detect and eliminate invading pathogens by inhibiting their replication and limiting host tissue damage. Through direct recognition of pathogen-associated molecular patterns and damage-associated molecular patterns, PRRs initiate inflammatory responses, including cytokine production, and modulate the adaptive immune response. Ligand binding activates downstream signaling pathways that promote pathogen clearance and drive inflammasome assembly. Accumulating evidence underscores the critical role of PRRs in sensing cellular damage and preserving homeostasis. Importantly, interactions within PRR networks facilitate the formation of multiple PRR-containing inflammasomes (PANoptosome), enabling coordinated inflammatory cell death under combined pathogen-associated molecular pattern and damage-associated molecular pattern stimulation. A comprehensive understanding of these interconnected signaling networks is essential for elucidating the regulation of innate immunity and its implications for disease pathogenesis, particularly in the context of infection and inflammation. This review provides a detailed overview of PRR-ligand recognition, downstream signaling mechanisms, and inflammasome activation, and discusses emerging insights into PRR regulation that hold promise for novel immunotherapeutic interventions.

## INTRODUCTION

Innate immunity serves as a critical first line of defense against pathogenic invasion, offering immediate and broad-spectrum protection to the host. This system operates through a combination of intrinsic barriers, including physical structures such as the skin and mucosal epithelium, as well as chemical factors like antimicrobial enzymes, pH regulation, and the complement cascade. Beyond these barriers, innate immune cells play a pivotal role by sensing conserved microbial components, referred to as pathogen-associated molecular patterns (PAMPs), as well as endogenous signals released from stressed or damaged cells, known as damage-associated molecular patterns (DAMPs). These molecular signatures are recognized by pattern recognition receptors (PRRs), which initiate downstream immune responses that contribute to pathogen control and the preservation of tissue integrity (Brubaker et al., 2015, Gong et al., 2020, Saïd-Sadier and Ojcius, 2012, Saijo et al., 2018).

**Fig. 1:**
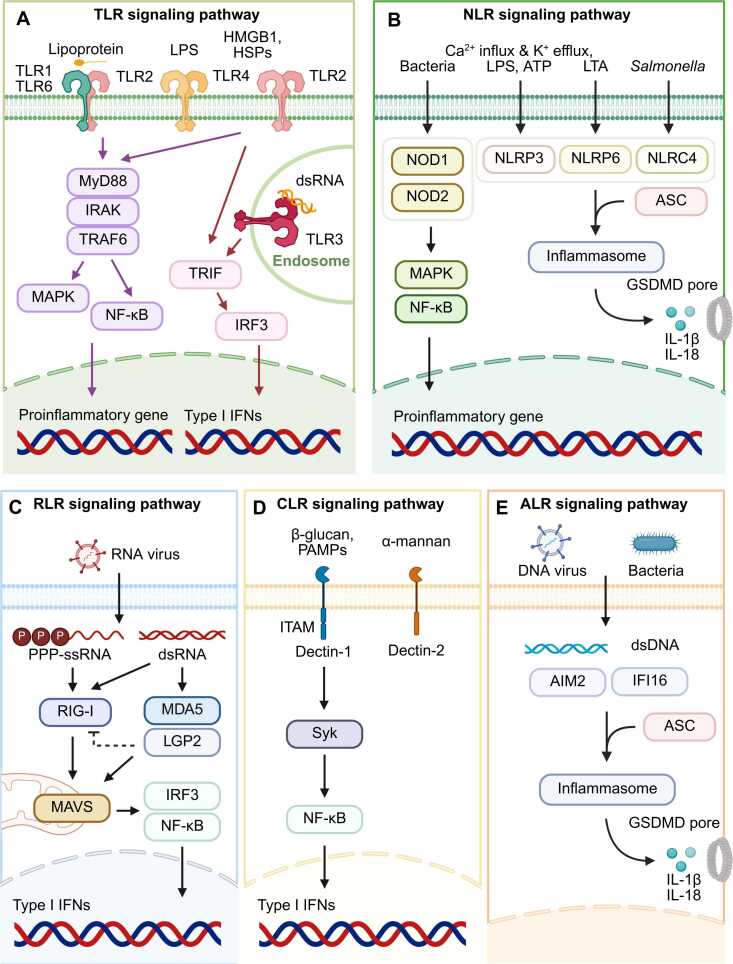
PRRs signaling pathway. PRRs are divided into 5 families: TLRs, NLRs, RLRs, CLRs, and ALRs. (A) TLRs can be located on plasma membrane or endosomal membrane, and recognize PAMPs (lipoprotein, LPS) and DAMPs (HMGB1, HSPs, and dsDNA), respectively. After recognition, they activate MyD88-dependent or MyD88-independent signaling pathway, resulting in the expression of proinflammatory genes and type I IFNs. (B) NLRs can recognize various DAMPs and PAMPs, such as LPS, ATP, and LTA, and lead to activation of proinflammatory genes. NLRs can form a protein complex, inflammasome. After the ligand recognition, they recruit the adapter protein, ASC, to form inflammasome and mediate the maturation of pore-forming protein, GSDMD, and proinflammatory cytokine, IL-1β. (C) RLRs can sense endogenous RNA and interact with MAVS, leading to activation of type I IFNs via IRF3-NF-κB axis. (D) Representative CLRs are Dectin-1 and Dectin-2. They recognize PAMPs, such as β-glucan and α-mannan. Dectin-1 has ITAM that induces Syk-mediated NF-κB activation. (E) ALRs can sense dsDNA and lead to inflammasome activation. GSDMD, gasdermin D; HMGB1, high-mobility group box 1; HSPs, heat shock proteins; LTA, lipoteichoic acid; MAPK, mitogen-activated protein kinase; PPP-ssRNA, triphosphate single-stranded RNA; Syk, spleen tyrosine kinase; TRIF, TIR domain–containing adapter-inducing IFN-β. Created with Biorender.com.

PRRs are broadly classified into 5 distinct families: Toll-like receptors (TLRs), nucleotide-binding oligomerization domain (NOD)-like receptors (NLRs), retinoic acid–inducible gene I (RIG-I)-like receptors (RLRs), C-type lectin receptors (CLRs), and absent in melanoma 2 (AIM2)-like receptors (ALRs) (Fig. 1). This classification reflects both their structural characteristics and subcellular localization. TLRs and CLRs function primarily as transmembrane receptors on the plasma membrane or within endosomal compartments, whereas NLRs, RLRs, and ALRs are cytosolic sensors. Upon direct engagement with PAMPs or DAMPs, PRRs initiate intracellular signaling cascades that culminate in the transcriptional activation of proinflammatory genes. Notably, several cytosolic PRRs also serve as key components in the assembly of inflammasomes—multiprotein complexes that orchestrate critical effector mechanisms of the innate immune response, including the maturation of inflammatory cytokines and the induction of pyroptotic cell death. Recent studies have revealed that PRRs can function cooperatively through both direct and indirect interactions, leading to the assembly of supramolecular signaling complexes termed PANoptosome. These complexes represent a higher-order organization of innate immune sensing, in which individual PRRs participate sequentially to regulate the activation of downstream inflammasome components. A representative example is Z-deoxyribonucleic acid (DNA)-binding protein 1 (ZBP1), which acts as an upstream regulator of the NOD-like receptor family pyrin domain (PYD)–containing 3 (NLRP3) inflammasome. During influenza A virus (IAV) infection, ZBP1 engagement facilitates the formation of a ZBP1-NLRP3 inflammasome complex, highlighting the functional convergence of distinct PRR signaling pathways in the context of antiviral innate immunity (Kuriakose et al., 2016, Malireddi et al., 2023, Oh et al., 2025). During Herpes simplex virus 1 (HSV-1) infection, AIM2 has been shown to transcriptionally regulate the expression of ZBP1 and pyrin, leading to the assembly of a composite AIM2-ZBP1-pyrin inflammasome (Lee et al., 2021b). This finding underscores the importance of inter-PRR interactions in shaping innate immune responses and highlights their potential as targets for therapeutic intervention. In this review, we summarize recent advances in our understanding of PRR activation and inflammasome assembly, with particular emphasis on the emerging concept of PANoptosome.

## TLRs

TLRs constitute a well-characterized family of PRRs that play a fundamental role in the early detection of pathogenic microorganisms. In humans, 10 functional TLRs (TLR1-TLR10) have been identified, whereas in mice, 12 functional members (TLR1-TLR9 and TLR11-TLR13) are present (Andrade et al., 2013, Lauw et al., 2005, Mathur et al., 2016, Temperley et al., 2008). Murine TLR10, however, is nonfunctional owing to the insertion of a reverse transcriptase element (Chuang and Ulevitch, 2001). TLRs are type I transmembrane glycoproteins and are classified based on their subcellular localization. TLR1, TLR2, TLR4-TLR6, and TLR11 (in mice) are expressed at the cell surface, where they primarily recognize microbial membrane components, while TLR3 and TLR7-TLR9 are localized to intracellular endosomal compartments, where they detect nucleic acids of microbial origin (Kawai and Akira, 2010). Each TLR shares a conserved structural organization, comprising an extracellular leucine-rich repeat domain responsible for ligand recognition, a single transmembrane domain, and an intracellular Toll/interleukin-1 (IL-1) receptor (TIR) domain that mediates signal transduction leading to the activation of downstream inflammatory pathways.

TLRs exhibit broad specificity for both PAMPs and DAMPs, thereby serving as essential mediators of innate immune activation. Among them, TLR2 forms functional heterodimers with either TLR1 or TLR6, facilitating the recognition of triacylated and diacylated lipopeptides, respectively (Farhat et al., 2008). TLR4, in collaboration with the accessory proteins myeloid differentiation factor 2 (MD2) and cluster of differentiation 14, detects lipopolysaccharide (LPS), a key component of Gram-negative bacterial cell walls. Cluster of differentiation 14 plays a crucial role in shuttling LPS to the TLR4-MD2 complex, where ligand engagement induces conformational rearrangements within the MD2 hydrophobic pocket, enabling receptor dimerization and downstream signal propagation (Miyake, 2007, Park et al., 2009). In addition to microbial ligands, TLR2 and TLR4 are also responsive to endogenous DAMPs such as high-mobility group box 1 and heat shock proteins, thereby linking infection-driven and sterile inflammatory responses (Ionita et al., 2010). TLR5, one of the most evolutionarily conserved members of the TLR family, recognizes bacterial flagellin—a function confirmed through crystallographic analysis in zebrafish (Yoon et al., 2012). Nonetheless, the structural and biochemical characterization of TLRs in nonmammalian species remains incomplete, limiting their translational applicability (Rout et al., 2021). Endosomal TLRs, including TLR3 and TLR7-TLR9, detect nucleic acid–derived PAMPs from viral and bacterial pathogens, and, under certain pathological conditions, also respond to endogenous nucleic acids (Akira et al., 2006). Although the precise biological function of TLR10 remains under investigation, recent evidence suggests its capacity to bind double-stranded ribonucleic acid (dsRNA) in an endosomal pH-dependent manner, implicating its role in antiviral defense (Lee et al., 2018b). In murine models, TLR11 and TLR12 mediate recognition of *Toxoplasma gondii* profilin (Hedhli et al., 2016, Kucera et al., 2010, Raetz et al., 2013).

TLR signaling is mediated through 2 pathways: the MyD88-dependent and the MyD88-independent pathways (Barton and Medzhitov, 2003, Shimizu, 2017). In the MyD88-dependent pathway, the adapter protein MyD88 binds to the intracellular TIR domain of activated TLRs. MyD88 then recruits IL-1 receptor-associated kinase 4 (IRAK4), which activates IRAK1 and IRAK2 through autophosphorylation. This leads to the formation of a complex with tumor necrosis factor (TNF) receptor–associated factor 6 (TRAF6), transforming growth factor-β-activated kinase 1 (TAK1), and TAK1-binding proteins (TAB1 and TAB4) (De Nardo et al., 2018, Gay et al., 2014, Häcker et al., 2006, Kawai and Akira, 2011, Verstak et al., 2009). The complex activates IκB kinase (IKK), which phosphorylates IκB, allowing nuclear factor κB (NF-κB) to enter the nucleus and induce the expression of proinflammatory genes such as IL-1, IL-6, and TNF (Cohen and Strickson, 2017, Kawai et al., 2004). In parallel, the IRAK1–TRAF6 axis activates mitogen-activated protein kinases, including p38 and c-Jun N-terminal kinase (JNK), to further support the inflammatory response (Alexopoulou et al., 2001, Hsu et al., 2007). The MyD88-independent pathway is associated with TIR domain–containing adapter-inducing interferon (IFN)-β. Upon activation, TIR domain–containing adapter-inducing IFN-β recruits TRAF3, IKKε, and TRAF family member-associated NF-κB activator (TANK)-binding kinase 1 (TBK1), 1, leading to phosphorylation of IFN regulatory factor 3 (IRF3) and the production of type I IFNs (Samie et al., 2018, Shalaby et al., 2017, Wu et al., 2019, Yamamoto et al., 2003) (Fig. 1A). Together, these signaling pathways enable TLRs to coordinate effective immune responses and maintain immune homeostasis.

## NLRs

NLRs constitute one of the largest families within the PRR superfamily. These cytosolic receptors detect a broad range of PAMPs and DAMPs, initiating downstream signaling pathways that modulate innate immune responses. To date, at least 23 functional NLRs have been identified in humans and 34 in mice. Structurally, NLRs are characterized by 3 conserved domains: an N-terminal effector domain, a central nucleotide-binding and oligomerization domain (NLR family apoptosis inhibitory proteins NOD domain), and a C-terminal leucine-rich repeat domain. Ligand recognition induces oligomerization of the NOD domain, facilitating the recruitment and assembly of downstream signaling complexes (Meunier and Broz, 2017).

Based on the nature of their N-terminal domains, mammalian NLRs are categorized into 5 subfamilies: the acidic transactivation domain–containing NLRs, baculoviral inhibitor of apoptosis repeat–containing NLRs (NLRB), caspase activation and recruitment domain (CARD)-containing NLRs (NLRC), NLRP, and the atypical group with undefined N-terminal motifs (NLRX) (Chou et al., 2023, Kim et al., 2016, Meunier and Broz, 2017, Ting et al., 2008, Velloso et al., 2019, Yeretssian, 2012).

Among the best-characterized NLRs are NOD1 (NLRC1) and NOD2 (NLRC2), which sense bacterial cell wall components—γ-D-glutamyl-meso-diaminopimelic acid (iE-DAP) and muramyl dipeptide, respectively (Chamaillard et al., 2003, Pashenkov et al., 2018). Upon activation, these receptors dimerize and recruit receptor-interacting serine-/threonine-protein kinase 2 (RIPK2) via CARD-CARD interactions (Park et al., 2007). RIPK2 subsequently activates TAK1, TAB1, and the IKK complex, resulting in the phosphorylation of IKKα/β and nuclear translocation of NF-κB, promoting the transcription of inflammatory genes (Hasegawa et al., 2008). Additionally, NOD1/2 signaling can activate mitogen-activated protein kinases, including p38 and JNK, further amplifying proinflammatory responses (Hsu et al., 2007).

A defining feature of several NLRs is their capacity to form inflammasomes that mediate caspase-1 activation and downstream pyroptotic cell death. The NLRP3 inflammasome, one of the most extensively studied, can sense a variety of stimuli, including microbial ligands (eg, LPS and adenosine triphosphate (ATP)) and ion fluxes such as K⁺ efflux and Ca²⁺ influx (Kayagaki et al., 2011, Lee et al., 2018a, Lee et al., 2019, Muñoz-Planillo et al., 2013, Pétrilli et al., 2007, Wang et al., 2022b). NLRC4 detects cytosolic flagellin from *Salmonella* (Karki et al., 2018, Zhao et al., 2011), whereas NLRP6 has been implicated in sensing lipoteichoic acid (Ghimire et al., 2018, Hara et al., 2018).

Upon activation, NLRs undergo conformational changes, exposing interaction motifs that facilitate recruitment of the adapter protein called apoptosis-associated speck-like protein containing as caspase recruitment domain (ASC) through homotypic PYD or CARD interactions (Cai et al., 2014, Lu et al., 2014, Masumoto et al., 1999). The resulting inflammasome complex activates caspase-1, which cleaves and activates gasdermin D (GSDMD), as well as the proinflammatory cytokines IL-1β and IL-18. Cleaved GSDMD forms membrane pores, enabling cytokine release and initiating pyroptosis—a lytic form of programmed cell death (Dinarello, 2009, Shi et al., 2015, Wang et al., 2021) (Fig. 1B).

## RLRs

RLRs are cytosolic PRRs that play a central role in antiviral immunity by sensing viral RNA. The RLR family comprises RIG-I, melanoma differentiation–associated gene 5 (MDA5), and laboratory of genetics and physiology 2 (LGP2) (Barral et al., 2009, Rehwinkel and Gack, 2020, Yoneyama et al., 2015). RIG-I and MDA5 share a common structural framework consisting of a central helicase domain, a C-terminal domain responsible for RNA binding, and 2 N-terminal CARDs that mediate downstream signaling. In contrast, LGP2 lacks CARDs and functions primarily as a regulator of RLR signaling. RIG-I also contains a unique repressor domain within its C-terminal domain that contributes to autoregulation and stabilization of RNA interactions (Brisse and Ly, 2019, Kang et al., 2002).

RIG-I recognizes short dsRNAs bearing 5′-triphosphate or 5′-diphosphate ends, as well as unmethylated 2′-O ribose groups (Cui et al., 2008, Devarkar et al., 2016, Lu et al., 2010, Zheng et al., 2018), and the helical domain contacts with the base-paired part of RNA (Jiang et al., 2011, Kowalinski et al., 2011, Luo et al., 2011). Upon RNA binding, RIG-I undergoes a conformational change that exposes its CARDs, enabling oligomerization. This process is stabilized by K63-linked polyubiquitination, which facilitates the interaction of RIG-I with mitocondrial antiviral signaling (MAVS), triggering downstream signaling cascades (Peisley et al., 2014, Zeng et al., 2010).

MDA5 primarily detects long dsRNA molecules, often produced during replication of RNA and DNA viruses. RNA immunoprecipitation sequencing has demonstrated that MDA5 binds viral RNAs during infections such as those caused by measles virus (Runge et al., 2014, Sanchez David et al., 2016). Additionally, LGP2 immunoprecipitation revealed that L-region antisense RNA from encephalomyocarditis virus is an MDA5 ligand (Deddouche et al., 2014). Despite this, the precise molecular mechanisms underlying MDA5-RNA interactions remain to be fully elucidated. LGP2 regulates both RIG-I and MDA5 activity in a context-dependent manner. It negatively modulates RIG-I signaling by interfering with RNA binding and suppressing type I IFN production. However, it can also promote MDA5 filament assembly and oligomerization, as observed during recognition of bell pepper endornavirus RNA (Duic et al., 2020). Following activation, both RIG-I and MDA5 engage MAVS, which is localized on the mitochondrial membrane. MAVS then recruits TRAF3, leading to the activation of TBK1 and IKKε kinases. These kinases phosphorylate IRF3 and IRF7, driving the expression of type I IFNs. Concurrently, NF-κB is activated, amplifying the proinflammatory and antiviral response (Goubau et al., 2013, Hartmann, 2017) (Fig. 1C).

## CLRs

CLRs are a family of PRRs characterized by the presence of a C-type lectin-like domain within their carbohydrate recognition domain, which facilitates the detection of carbohydrate structures on both self and non–self entities (Dam and Brewer, 2010, Lan et al., 2020, Lepenies et al., 2013). CLRs are predominantly expressed on antigen-presenting cells such as dendritic cells (DCs) and macrophages (Ebner et al., 2003). Notable members of this family include DC–associated C-type lectin-1 (Dectin-1) and Dectin-2 (Kaisar et al., 2018, Yamaguchi et al., 2021). Dectin-1 is a type II transmembrane CLR that contains an immunoreceptor tyrosine-based activation motif (ITAM) in its cytoplasmic tail (Liu et al., 2015). It is primarily expressed in innate immune cells, including DCs and macrophages (de Quaglia et al., 2019), and plays a critical role in the recognition and clearance of PAMPs via complement activation. Upon binding its ligand β-1,3-glucan, Dectin-1 activates downstream signaling through both tyrosine kinase–dependent and tyrosine kinase–independent mechanisms (Zhou et al., 2018). Specifically, ITAM facilitates the recruitment and activation of spleen tyrosine kinase, leading to NF-κB activation (Rogers et al., 2005, Ye et al., 2020). In contrast, Dectin-2 lacks a cytoplasmic ITAM motif and relies on associated signaling adapters to mediate its effects. It primarily recognizes α-mannan structures present in the fungal cell wall (Ambati et al., 2019, Saijo et al., 2010). Functional recognition by Dectin-2 requires (α1→2)-linked mannosides, as demonstrated using purified mannose-capped lipoarabinomannan from *Mycobacterium tuberculosis* (Feinberg et al., 2017). Moreover, Dectin-2 can detect bacterial lipoglycans, emphasizing its broader role in microbial surveillance (Feinberg et al., 2017, Wittmann et al., 2016). Despite the absence of intrinsic signaling domains, Dectin-2 engages downstream pathways through multivalent interactions and accessory molecules, highlighting its essential function in CLR-mediated immunity (Decout et al., 2018) (Fig. 1D).

## ALRs

ALRs are a class of PRRs sensing intracellular DNA (Feng et al., 2019). Structurally, ALRs are characterized by a PYD at the N-terminus and a hematopoietic IFN-inducible nuclear protein with a 200–amino acid repeat (HIN200) domain at the C-terminus. The HIN200 domain mediates the recognition and binding of cytosolic dsDNA, while the PYD facilitates downstream signaling through interaction with the adapter protein (Hornung et al., 2009, Man and Kanneganti, 2015, Man et al., 2016, Wang et al., 2019). To date, 4 proteins have been classified as members of the ALR family: AIM2, IFN gamma-inducible protein 16 (IFI16), IFN-inducible protein X, and myeloid cell nuclear differentiation antigen (Caneparo et al., 2018, Jiang et al., 2021). Among them, AIM2 is the most well-characterized and plays a central role in recognizing dsDNA from various sources, including viruses, bacteria, synthetic analogs, and host-derived DNA. Upon DNA binding, AIM2 oligomerizes along the DNA strand and forms the AIM2 inflammasome, initiating caspase-1 activation and pyroptosis (Cadena and Hur, 2019, Lee et al., 2021b, Sung et al., 2012, Wang et al., 2022b). IFI16 also serves as a sensor of intracellular DNA and contributes to innate immune activation by inducing the expression of IFNs and other inflammatory mediators (Unterholzner et al., 2010, Wang et al., 2018) Despite differences in subcellular localization and response mechanisms, both AIM2 and IFI16 exemplify the essential role of ALRs in host defense against pathogenic DNA (Fig. 1E).

## NON-NLR PROTEINS: ZBP1 AND PYRIN

ZBP1 is an essential cytosolic inflammasome sensor. ZBP1 contains 2 Zα domains (Zα1 and Zα2) for binding its ligand, Z-form nucleic acid, and a receptor-interacting protein homotypic interaction motif (RHIM) for direct interaction with other RHIM-containing proteins, such as RIPK3 and RIPK1, leading to the formation of ZBP1 inflammasome (Ha et al., 2008, Kuriakose et al., 2016, Maelfait et al., 2017, Thapa et al., 2016, Wang et al., 2022b). ZBP1 has been shown to play a pivotal role in host defense against RNA viruses, including IAV and severe acute respiratory syndrome coronavirus 2 (SARS-CoV-2), where it drives inflammatory cell death pathways. Moreover, ZBP1 activation has been linked to cellular stress induced by nuclear export inhibition, further highlighting its role in maintaining cellular homeostasis (Karki et al., 2022, Karki et al., 2021, Kuriakose et al., 2016, Malireddi et al., 2023, Thapa et al., 2016).

Pyrin represents another well-characterized cytosolic inflammasome sensor that responds to disturbances in cytoskeletal dynamics, particularly through the inactivation of Rho GTPases by bacterial toxins. This event triggers the assembly of the pyrin inflammasome, also referred to as PYD and CARD domain- containing (PYCARD) inflammasome (Dumas et al., 2014, Xu et al., 2014). Pyrin comprises an N-terminal PYD, 2 B-box domains, a coiled-coil domain, and, uniquely in humans, a C-terminal B30.2 domain (Broz and Dixit, 2016). Upon activation, pyrin engages the adapter protein ASC via PYD-PYD homotypic interactions, facilitating the recruitment and activation of caspase-1. This leads to the proteolytic maturation of proinflammatory cytokines such as IL-1β, thereby promoting downstream inflammatory responses (Srinivasula et al., 2002).

## PRR-PRR CROSSTALK AND PANOPTOSOME

Inflammasomes are critical signaling platforms in innate immunity that orchestrate host responses to pathogens and cellular stress. These complexes can be assembled via canonical and noncanonical pathways, primarily through homotypic and heterotypic domain interactions. Canonical inflammasome activation is initiated upon sensing PAMPs or DAMPs by PRRs, leading to caspase-1 activation, processing of IL-1β and IL-18, and induction of pyroptosis. PRRs involved in canonical inflammasomes include NLRP1, NLRP3, NLRP6, NLRC4, ZBP1, and pyrin. Noncanonical pathways, mediated by caspase-4 and caspase-5 in humans and caspase-11 in mice, also contribute to inflammatory responses (Kayagaki et al., 2015, Matikainen et al., 2020).

**Fig. 2:**
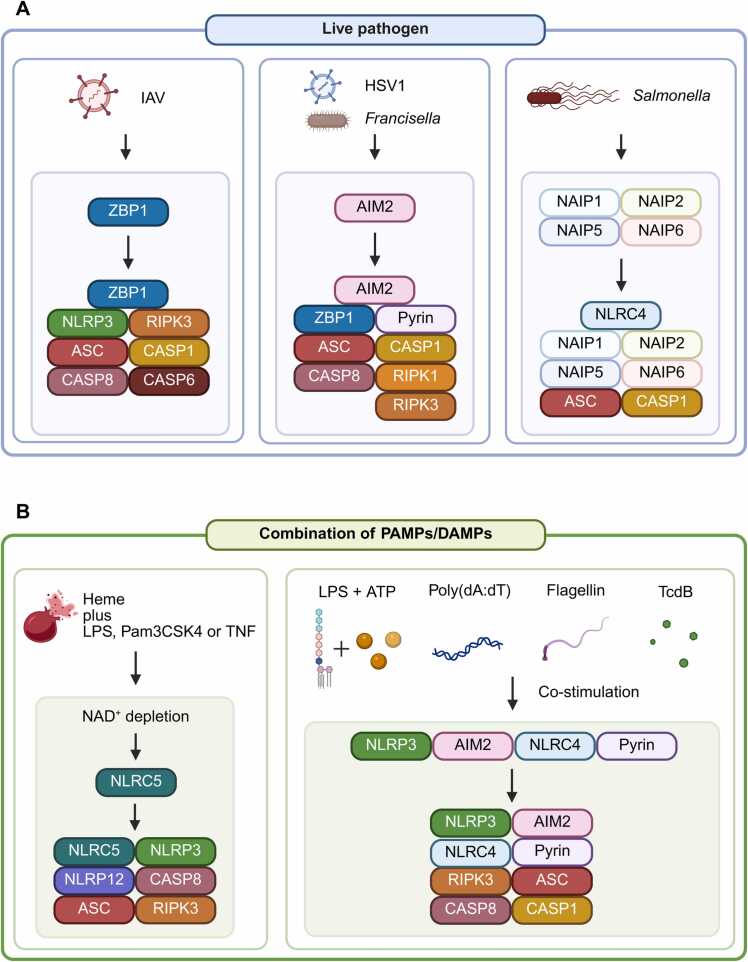
PANoptosome. (A) Live pathogens, such as viruses and bacteria, contain various PAMPs. When pathogens infect our cells, PRRs are exposed by multiple PAMPs and induce immune responses simultaneously. For instance, IAV infection is firstly recognized by ZBP1 and ZBP1 recruits NLRP3 and other inflammasome components. For HSV-1 and *Francisella* infection, AIM2 senses pathogen infection and induces ZBP1 and pyrin activation, resulting in multiple inflammasomes. *Salmonella* infection induces NAIPs and activates NLRC4 following the formation of NLRC4 inflammasome. (B) The combination of DAMPs and PAMPs can induce multiple inflammasomes. Heme plus PAMPs (LPS and Pam3CSK4) or TNF activate NLRC5, leading to inflammasome with NLRP12 and NLRP3. Co-treatment with LPS + ATP, Poly(dA:dT), Flagellin, and TcdB stimulates their corresponding PRRs, and eventually induces multiple PRR-containing inflammasomes. CASP, Caspase; NAIP, NLR family apoptosis inhibitory proteins. Created with Biorender.com.

Importantly, emerging evidence indicates that inflammasomes are not always formed by a single PRR acting in isolation. Instead, multiple PRRs can dynamically interact, co-regulate one another’s expression and activity, and co-assemble into a composite inflammasome complex amplifying the inflammatory response, termed PANoptosome (Lee et al., 2021b, Malireddi et al., 2023, Oh et al., 2023, Sundaram et al., 2023, Yu et al., 2024) (Fig. 2). While previous studies typically explored one-to-one PRR-PAMP interactions, recent findings reflect a more integrated picture: during infection by complex pathogens, which carry multiple PAMPs, or under stress conditions, co-activation and crosstalk among different PRRs result in the formation of multiple PRR-containing PANoptosomes that initiate diverse inflammatory cell death: Pyroptosis, Apoptosis, and Necroptosis (PANoptosis).

ZBP1 exemplifies this principle as a central hub during RNA virus infections, including IAV and SARS-CoV-2, where it cooperates with NLRP3 to form PANoptosome, driving robust inflammation and cell death (Karki et al., 2022, Kuriakose et al., 2016, Malireddi et al., 2023). ZBP1 can also sense disrupted cellular homeostasis, as shown in nuclear export inhibition, where its interaction with NLRP3 potentiates inflammasome activation (Karki et al., 2021). Furthermore, ZBP1 can be transcriptionally induced by AIM2 and incorporated into PANoptosome during Herpes simplex virus 1 infection, along with pyrin, highlighting a functional and physical network of PRR interactions that converge on inflammasome signaling (Lee et al., 2021b). Other examples include *Salmonella* infection, where the NLR family of apoptosis inhibitory proteins recognizes bacterial components and recruits NLRC4 for inflammasome assembly (Karki et al., 2018, Kofoed and Vance, 2011, Zhao et al., 2011) (Fig. 2A), and the recently discovered NLRP12-NLRP3 PANoptosome, which mediates PANoptosis in response to heme and additional stimuli (LPS, Pam3CSK4, or TNF) (Sundaram et al., 2023). Interestingly, NLRC5 was shown to partner with NLRP12 when activated by nicotinamide adenine dinucleotide (NAD^+^) depletion and TLR2/4 signals, further expanding the paradigm of PRR collaboration in inflammasome biology (Sundaram et al., 2024).

A particularly compelling demonstration of synthetic inflammasome synergy comes from Oh et al. (2023), who showed that simultaneous stimulation of 4 distinct PRRs—NLRP3, AIM2, NLRC4, and pyrin—leads to the formation of multi-PRR-containing PANoptosomes. This complex mediated robust inflammatory cell death and cytokine release, far exceeding the effect of individual PRR activation (Fig. 2B). This finding underscores a key emerging concept: simultaneous activation of multiple PRRs not only coexists but converges, coordinating to form structurally and functionally integrated inflammasomes.

Despite recent advances, the precise mechanisms underlying the coordination among different PRRs remain incompletely understood. In particular, how upstream PRRs influence the expression and activation of downstream PRRs, and how these sensors are spatially and temporally integrated into unified inflammasome complexes, are important questions yet to be fully resolved. Elucidating these regulatory networks will be crucial for advancing our understanding of inflammasome biology and may provide new opportunities for therapeutic intervention in inflammatory and infectious diseases.

## CONCLUSION AND PERSPECTIVE

PRRs are central to the innate immune system, acting as sentinels that detect pathogenic threats and cellular stress to initiate appropriate inflammatory responses. They not only trigger signaling pathways such as NF-κB and type I IFN but also assemble inflammasomes that mediate pyroptosis and the release of proinflammatory cytokines. This dual role enables PRRs to both contain infections and maintain immune homeostasis. However, dysregulated PRR activity can exacerbate inflammation and contribute to the development of inflammatory and autoimmune diseases (Karki et al., 2022, Lee et al., 2020, Lee et al., 2021a, Oh and Lee, 2023, Wang et al., 2022a), highlighting the need for tightly controlled PRR signaling.

A significant advancement in this field is the recognition of PANoptosomes, which are formed through the coordinated activation of distinct PRRs in response to complex stimuli (Karki et al., 2018, Karki et al., 2022, Kofoed and Vance, 2011, Kuriakose et al., 2016, Lee et al., 2021b, Malireddi et al., 2023, Oh et al., 2023, Place et al., 2021, Sundaram et al., 2024, Sundaram et al., 2023, Zhao et al., 2011). Unlike the classical view of single PRR-driven responses, this emerging concept emphasizes the combinatorial nature of innate sensing, where interactions between PRRs, such as ZBP1, NLRP3, AIM2, NLRC4, and pyrin, amplify immune responses and drive PANoptosis. PANoptosomes are not only activated by live pathogens but can also be experimentally induced by the co-administration of specific DAMPs and PAMPs, demonstrating the intricate interconnectedness of the PRR network.

Despite the growing understanding of these complexes, the mechanisms by which multiple PRRs are recruited, assembled, and regulated within PANoptosome remain incompletely defined. Key questions include how the temporal sequence of PRR activation shapes the resulting immune response, what scaffolding or adapter proteins facilitate their interaction, and how different cell types orchestrate these events under stress conditions.

A comprehensive understanding of these multi-PRR networks is essential for uncovering how innate immunity is fine-tuned in both protective and pathological contexts. Elucidating these mechanisms will not only provide insight into the fundamental biology of host defense but also open new avenues for therapeutic strategies aimed at modulating inflammasome activity in infectious, inflammatory, and immune-mediated diseases.

## Funding and Support

This work was supported by the National Research Foundation of Korea (10.13039/100007431NRF) grant funded by the Korea government (MSIT) (2022R1C1C1007544, 2024M3A9H5043152 to S.L.), a grant from 10.13039/501100004080Korea Drug Development Fund funded by Ministry of Science and ICT, Ministry of Trade, Industry, and Energy, and Ministry of Health and Welfare (RS-2025-02222987 to S.L.), a grant from the Korea Health Industry Development Institute (10.13039/501100003710KHIDI) funded by the Ministry of Health & Welfare, Republic of Korea, under the Korea Health Technology R&D Project (RS-2022-KH128422(HV22C015600) to S.L.), and the Institute for Basic Science (10.13039/100011290IBS), Republic of Korea (IBS-R801-D1 to Y.K.C, IBS-R801-D9-A09, and IBS-R801-D1-2025-a02 to S.L.). Moreover, this work was supported by The Circle Foundation (Republic of Korea) through the selection of the UNIST Pandemic Treatment Research Center as the 2023 The Circle Foundation Innovative Science Technology Center (2023 TCF Innovative Science Project-01 to S.L.). Additionally, this study received funding from the Republic of Korea’s National Institute of Health (Project No. #2025ER160200, #2025ER240100 to S.L.). Additional support was provided by research funds from Ulsan National Institute of Science & Technology (10.13039/501100002613UNIST) (1.220112.01, 1.220107.01 to S.L.), The Korean Society of Ginseng 2023 (S.L.), and a grant from Yuhan Corporation (S.L.).

## Author Contributions

Young Ki Choi: Writing—review and editing, Funding acquisition, and Conceptualization. SangJoon Lee: Writing—review and editing, Writing—original draft, Validation, Supervision, Funding acquisition, and Conceptualization. SuHyeon Oh: Writing—review and editing, Writing—original draft, Conceptualization.

## Declaration of Competing Interests

The authors declare that they have no known competing financial interests or personal relationships that could have appeared to influence the work reported in this paper.
